# Pivoting between Calmodulin Lobes Triggered by Calcium in the Kv7.2/Calmodulin Complex

**DOI:** 10.1371/journal.pone.0086711

**Published:** 2014-01-28

**Authors:** Alessandro Alaimo, Araitz Alberdi, Carolina Gomis-Perez, Juncal Fernández-Orth, Ganeko Bernardo-Seisdedos, Covadonga Malo, Oscar Millet, Pilar Areso, Alvaro Villarroel

**Affiliations:** 1 Unidad de Biofísica, CSIC, UPV/EHU, Universidad del País Vasco, Leioa, Spain; 2 Departamento de Farmacología, UPV/EHU, Universidad del País Vasco, Leioa, Spain; 3 Structural Biology Unit, CICbioGUNE, Bizkaia Technology Park, Derio, Spain; Cinvestav-IPN, Mexico

## Abstract

Kv7.2 (KCNQ2) is the principal molecular component of the slow voltage gated M-channel, which strongly influences neuronal excitability. Calmodulin (CaM) binds to two intracellular C-terminal segments of Kv7.2 channels, helices A and B, and it is required for exit from the endoplasmic reticulum. However, the molecular mechanisms by which CaM controls channel trafficking are currently unknown. Here we used two complementary approaches to explore the molecular events underlying the association between CaM and Kv7.2 and their regulation by Ca^2+^. First, we performed a fluorometric assay using dansylated calmodulin (D-CaM) to characterize the interaction of its individual lobes to the Kv7.2 CaM binding site (Q2AB). Second, we explored the association of Q2AB with CaM by NMR spectroscopy, using ^15^N-labeled CaM as a reporter. The combined data highlight the interdependency of the N- and C-lobes of CaM in the interaction with Q2AB, suggesting that when CaM binds Ca^2+^ the binding interface pivots between the N-lobe whose interactions are dominated by helix B and the C-lobe where the predominant interaction is with helix A. In addition, Ca^2+^ makes CaM binding to Q2AB more difficult and, reciprocally, the channel weakens the association of CaM with Ca^2+^.

## Introduction

Calmodulin (CaM) orchestrates cell function by interacting with a large number of different proteins, conferring them Ca^2+^-dependent regulation [1]; [2]. CaM is composed of two homologous domains (the N-terminal and C-terminal domains), commonly referred to as lobes. The N-lobe and the C-lobe are tethered by a short and flexible linker, and each domain contains a pair of helix-loop-helix motifs, known as the EF-hands (Ca^2+^ binding motifs). The Ca^2+^ affinity for EF3 and EF4 in the C-lobe is higher than that of sites EF1 and EF2 in the N-lobe [3], although this changes upon complexing with a target [4]; [5]. Binding can reciprocally affect the conformation of CaM and its target, consequently changing the complex’s affinity for Ca^2+^ [1]; [6].

The lobes of CaM are relatively rigid when Ca^2+^ is bound, although segmental inter-domain motion has been described [7]. Such structural flexibility explains how CaM is capable of interacting with target proteins with different structural features [8]. Globally, conformational changes of CaM include at least two major events: the unwinding of the alpha-helix in the linker region, and exposure of the hydrophobic interfaces of both the N- and C-lobes upon Ca^2+^-association. The flexibility of the central region permits CaM to readily alter its domain orientation, allowing it to adopt a range of conformations from a compact (C shape) to a fully extended conformation (S shape) [6]. In the absence of Ca^2+^, the two lobes of CaM adopt a S or C shape in a “closed conformation” in which a bundle of helices are well packed against each other, and most of the hydrophobic residues are shielded from the solvent. Upon Ca^2+^ binding, CaM changes its conformation from the closed configuration to an open one, exposing the hydrophobic surfaces within the N- and C-lobes for Ca^2+^-dependent interactions with target proteins [8]. The structural malleability of CaM is extraordinary, as highlighted by the complexes with two variants of the SK2 potassium channel, where different sets of amino acid residues contribute to the hydrophobic interface at CaM, even though the interacting amino acids on the targets remain the same [6]; [9].

Several targets can interact to different extents with CaM in its Ca^2+^-free (apo-CaM) and Ca^2+^-bound (holo-CaM) states, thereby generating a range of Ca^2+^-dependent responses [1]. Some proteins bind more favorably to the N-lobe and other to the C-lobe, whereas in proteins like Kv7 (KCNQ) potassium channels, both CaM domains are partners in the processes of association and function [10]. Kv7.2 and Kv7.3 are expressed in the nervous system and they are the principal molecular components of the slow voltage gated M-channel that exerts an important influence on neuronal excitability [11]. CaM has been proposed to be essential for tetramerization of Kv7.1 channels [12]; [13], but not for Kv7.2 subunits where CaM is crucial for the channels to exit the ER, and to mediate Ca^2+^-dependent inhibition [14]–[17]. Indeed, mutations that disrupt CaM binding to helix A are linked to Benign Familial Neonatal Convulsions (BFNC) [18]; [19]. Like all Kv channels, the Kv7 alpha subunits share a common core structure composed of six transmembrane segments with a voltage-sensing domain (S1–S4), a pore domain (S5–S6) and intracellular N- and C-terminal regions [20]. CaM binds simultaneously to two sites of Kv7.2 in a 1∶1 stoichiometry [21]; [22]. The sites are located in the intracellular C-terminus at 30–40 Å from the mouth of the pore, where the N-lobe is closer to the conduction pathway [23]. These two sites are thought to adopt an alpha helical configuration, and they are referred to as helices A and B [10]; [22]. The binding of CaM is transient [23]; [24], and it can occur on the same or different subunits of the tetrameric channel [25]. Both isolated segments can bind CaM in the presence of Ca^2+^, while in its absence, an association with each individual segment is difficult to detect [10]; [25]. By contrast, apo-CaM interacts readily with the complete CaM binding domain, although the basis of this particular behavior is not understood.

The structure of helix B from Kv7.4 complexed with holo-CaM was recently resolved [22], although the association of CaM with the Kv7.2 binding domain clearly differs from its interaction with the individual segments [25]. Although helix A, an essential component of the Kv7 CaM binding domain, is not present in the resolved complex, this structure provides valuable information on how CaM interacts with the channel. However, the molecular details of how Ca^2+^ triggers its regulatory effects on the M-current are unknown. Here, we have studied this functional interaction using fluorescence and NMR spectroscopy, in order to compare the association between Kv7.2 and full-length CaM 1–148 with that between Kv7.2 and the isolated N- (CaM 1–78) and C-domain (CaM 79–148) fragments [7]. By comparing the residues affected by these interactions, as seen by NMR, and known disease causing mutations, we elaborate a model of the Ca^2+^-dependent regulation.

## Experimental Procedures

### Purification of Recombinant Proteins

The Kv7.2 CaM binding domain (residues 310–548 from human Kv7.2 Y15065), named here Q2AB WT and the R353G and L339R mutants, helix A (hA residues G310–T359) and helix B (hB residues S450–S590), fused to GST, were expressed in *E. coli* BL21–DE3 cells and purified using GSH sepharose as described previously [26]. Recombinant rat brain CaM (NM_017326) was produced in BL21–DE3 bacteria and purified as described [27]. The rat KCa2.2 (SK2) CaM binding domain (residues 395–490) cDNA (NM_019313) was kindly provided by John P. Adelman (Vollum Institute). This protein contains a C-terminal 6xHis tag and was purified as described [9]. The cDNAs encoding the human CaM N-lobe (residues 1–78) and C-lobe (residues 79–148) were provided by the group of Daniel L. Minor Jr. (Cardiovascular Research Institute, University of California) and both lobes were produced as described previously [28]. There are no differences in the primary amino acid sequence of rat and human CaM. CaM12 and CaM34 were a generous gift from Katalin Török (St. George’s University of London) [29]. CaM12 has two alanine substitutions in the first and second EF hands (D22A, D58A) while CaM34 has mutations in the EF3 and EF4 (D95A, D131A). The oligomerization state of the purified proteins was examined by dynamic light scattering (DLS) using a Zetasizer Nano instrument (Malvern Instruments Ltd.) and by size exclusion chromatography. Further details on purification procedure and on quality control can be found in Supporting Material in File S1.

### Fluorescence Spectroscopy

In the fluorometric experiments using dansyl-CaM (D-CaM), the CaM dansylation, sample preparation and fluorescent measurements were performed as described [26]. The D-CaM binding buffer used was: 25 mM Tris-HCl, 120 mM KCl, 5 mM NaCl, 2 mM MgCl_2_, 10 mM EGTA, pH 7.4). The level of contaminant Ca^2+^ in the protein preparations was determined by inductively coupled plasma mass spectroscopy carried out at the Department of Analytical Chemistry (University of the Basque Country), and was found to be less than 40 nM. Experiments were also performed in the presence of an excess of free Ca^2+^ (3.9 or 100 µM), adding 9.63 or 9.985 mM Ca^2+^ to the D-CaM binding buffer. The data obtained at lower (3.9 µM) and higher (100 µM) free Ca^2+^ concentration were indistinguishable. Fluorescence enhancement was plotted against that in [Ca^2+^ free] to generate the concentration-response curves. The parameters of the Hill equation (E = Bmax*([Ligand]^h^/([Ligand]^h^+IC_50_^h^)), or the parameters of a two sites Hill equation (E = Bmax_1_*([Ligand]^h1^/([Ligand]^h1^+ IC_50_1^h1^))+Bmax_2_*([Ligand]^h2^/([Ligand]^h2^+ IC_50_2^h2^)) were fitted to the data by curvilinear regression, enabling the apparent affinity (EC_50_ or concentration that gives half-maximal change in fluorescence emission intensity) and Hill coefficient to be determined.

In the competition assays, the purified CaM N-, and C-lobe, and an equimolar mixture of both lobes or intact CaM were added in increasing amounts to the starting mixture of D-CaM (12.5 nM) and a ligand (Q2AB, SK2, hA or hB). Steady state fluorescence was reached within 30 seconds, and measurements were taken more than 60 seconds after sample addition. No significant effect of the isolated lobes or GST was found. The reduction in fluorescence was plotted against the concentration of the lobes. The concentration of purified CaM, CaM12 and CaM34 was determined by absorbance. In the competition assays with the SK2 protein we found that the results obtained with the isolated N-lobe were comparable to those obtained with CaM34 (CaM34 is a mutated CaM in which Ca^2+^ does not bind to the C-lobe). Similarly, CaM12 behaved like the isolated C-lobe. Finally, the inhibition curves were used to calibrate the concentration of the isolated lobes (see Fig. S4 in File S1). The concentrations of the GST fusion proteins, SK2 and the pA synthetic peptide were determined by using their intrinsic UV absorption.

To make the assay more sensitive we used the concentration of the ligand that corresponded to its calculated EC_50_ for the increase in D-CaM fluorescence emission [26]. The data was plotted as the reduction in fluorescence (taking the initial complex as 100%) relative to the [competing lobe/s]. The following equation, which assumes as 1∶1 stoichiometry, was used to estimate the K_d_, where F is the increase in fluorescence, F_max_ is a free parameter that represents the maximal fluorescence, [peptide] is the known total peptide concentration, [CaM] is the known concentration of total D-CaM, and K_d_ is a free parameter for the affinity constant:



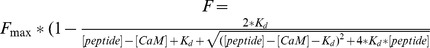
The results are expressed as the means ± S.E.M from three or more experiments. For statistical comparison, significance was evaluated using the unpaired Student t test, with values of P < 0.05 (*), P < 0.01 (**) and P < 0.001 (***) considered to be statistically significant.

### NMR Measurements

Uniformly ^15^N-labeled CaM was prepared in M9 medium containing 1 g/L ^15^NH_4_Cl as source of nitrogen. Other steps involved in the expression and purification of ^15^N-labeled CaM were the same as for WT CaM. The ^2^D-^1^H,^15^N-HSQC experiments were performed by dissolving CaM (25–50 µM) in a buffer consisting of 50 mM Tris-HCl, 100 mM KCl, 10% D_2_O and 10 mM EGTA (apo-CaM) or, alternatively, 5 mM CaCl_2_ (holo-CaM).The pH values of the sample solutions were carefully adjusted to 7.4 with trace amount of 2 M KOH. All NMR experiments were carried out at 25°C on a Bruker Avance III 600 MHz NMRs spectrometer. The HSQC spectra were acquired with a spectral width of 30 ppm in the ^15^N dimension and 16 ppm in the ^1^H dimension. In chemical shift perturbation (CSP) experiments, peptide-CaM complexes were prepared by adding 1–10 equivalents of the appropriate lyophilized peptide (Q2AB WT, Q2AB R353G, Q2AB L339R or pA peptide) directly to the CaM NMR sample. The CSP studies were performed by monitoring the changes in the ^1^H,^15^N HSQC spectra of ^15^N-labeled CaM. The CSP values were then evaluated as a weighted average chemical shift difference of ^1^H and ^15^N resonances, using the equation:
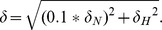



## Results

We used two complementary approaches to characterize the interaction of CaM with Kv7.2 (KCNQ2), the first monitored the fluorescence changes of the dansyl group covalently bound to CaM (D-CaM) in an assay that is very sensitive and requires low protein concentrations (6.25–200 nM). D-CaM reports the binding to target peptides or Ca^2+^ based on the fluorescence enhancement when the environment of the dansyl group becomes hydrophobic [30]; [31]. No differences have been found between the interactions of normal and dansylated CaM derivates with several ligands [32]. In addition, NMR experiments identify residues affected by the interaction that are not necessarily in direct contact with the ligand (see below).

**Figure 1:**
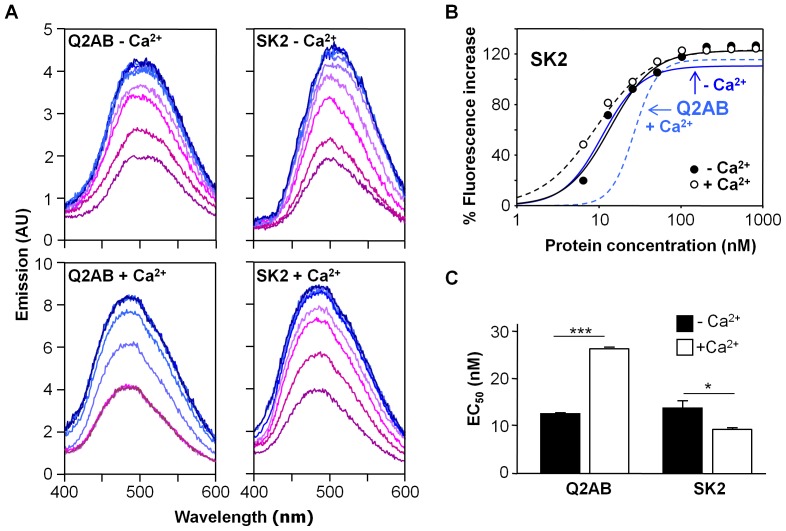
Dose-response enhancement of 12.5 nM D-CaM fluorescence emission by the CaMBD. *(A)* Effect of an incremental addition of the Q2AB (left column) or SK2 CaM binding domains (right column) in the emission spectra of 12.5 nM D-CaM both in the absence of free Ca^2+^ (top panels, 10 mM EGTA added) and in the presence of 3.9 µM free Ca^2+^ (bottom panels). The color of the traces changes from red to blue as the ligand concentration increases. *(B)* Relative concentration-dependent enhancement of 12.5 nM D-CaM fluorescence emission by SK2 in the presence (open circles) or absence (filled circles) of 3.9 µM Ca^2+^. The parameters used to fit a Hill equation to the data (continuous and dashed lines) were: Max = 122±4.3, EC_50_ = 13.7±1.6 nM, h = 1.6±0.3 in absence of Ca^2+^, and Max = 123±1.4, EC_50_ = 9.2±0.4 nM, h = 1.3±0.1 in the presence of Ca^2+^. The data represent the means ± standard error from three or more independent experiments. The error bars are smaller than the symbols. For comparison, the result of the fit of a Hill equation to the data for the effect of Q2AB of D-CaM fluorescent emission taken from [26] is plotted in grey in the absence (continuous grey line) or presence of Ca^2+^ (dotted grey line). *(C)* Plot of the apparent binding affinity derived from the data in B obtained in absence (black column) or in presence of Ca^2+^ (white columns) for the proteins indicated. ***, significance at P≤0.001, *P≤0.05, unpaired Student’s t test.

Two discontinuous segments constitute the CaM-binding site (CaMBD) of Kv7.2 channels [10], which is reminiscent of the two alpha helices in the KCa2.2 potassium channel CaM-binding site (SK2). We compared the binding behavior of the SK2 CaMBD to that of the Kv7.2 CaMBD (Q2AB), as characterized previously [26]. The intensity of the fluorescence emission of D-CaM augmented with the concentration of the different peptides (Fig. 1 *A*). In contrast to Ca^2+^, that causes an increase in the fluorescence emission of D-CaM, no blue-shift in the emission peak was observed for any of the peptides analyzed in this study, ruling out the existence of contaminating calcium. Dose-response curves were constructed from these data in the absence of Ca^2+^ (10 mM EGTA added) and in the presence of 3.9 µM free Ca^2+^ (9.63 mM Ca^2+^, 2 mM Mg^2+^ and 10 mM EGTA, pH 7.4), establishing the apparent affinities that represent an upper limit for the intrinsic K_d_ value (Fig. 1 *B* and 1 *C,* see Fig. S5 in File S1). The maximal increase of D-CaM fluorescence emission induced by a molar excess of SK2 and Q2AB were comparable for the holo and apo states (Fig. 1 *A* and *B*). The apparent binding affinity in the absence of Ca^2+^ was similar for SK2 and Q2AB (Fig. 1). However, the presence of free Ca^2+^ increased the apparent affinity for SK2 (from 13.7±1.6 nM to 9.2±0.4 nM) while decreasing that for Q2AB (from 11.0±0.5 nM to 27.1±0.1 nM, Fig. 1 *C*), revealing substantial differences in CaM binding to both targets.

### The Interaction with SK2 is Dominated by the C-lobe in the Absence of Calcium Whereas the Affinity for the N-lobe Increases in the Presence of this Ion

**Figure 2:**
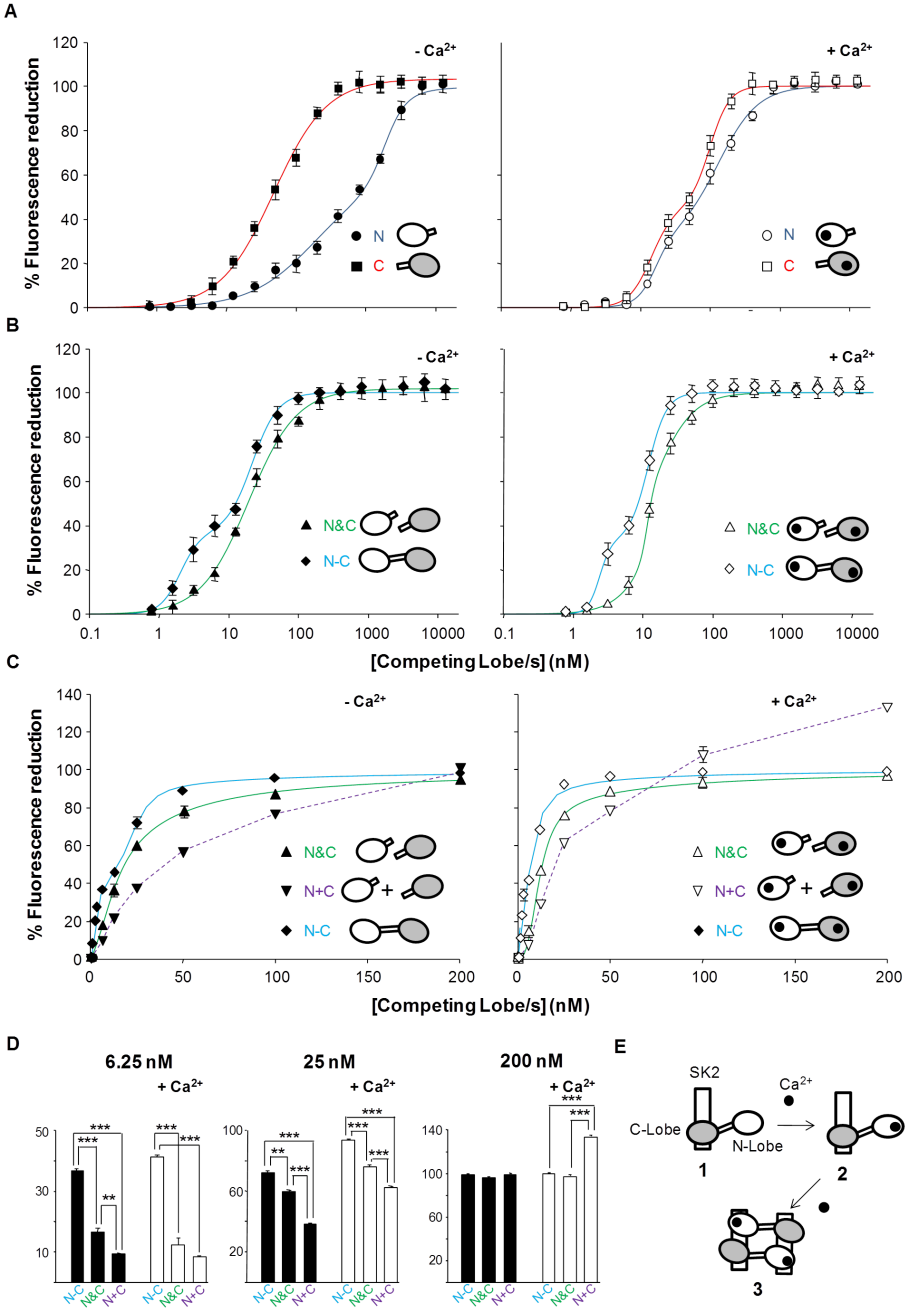
The competition assay defines lobe specific interactions with SK2. Competition curves with isolated CaM lobes (N or C), with an equimolar mixture of N- and C-lobes (N&C) or with intact CaM (N–C). D-CaM (12.5 nM) was mixed with SK2 at a concentration corresponding to its calculated EC_50_ for the increase D-CaM fluorescence emission (see Fig. 1 *C*, 9.2 and 13.7 nM in the presence or absence of Ca^2+^, respectively) and the competing peptides were added incrementally at the concentrations indicated. The data represent the means ± standard error from three or more independent experiments. The error bars are smaller than the symbols. The result of fitting a Hill equation to the competition curves is compiled in Table 1. *(A)* The effect of incremental addition of the lobes indicated obtained in the absence of Ca^2+^ (Left, 10 mM EGTA added) and in the presence of 100 µM free Ca^2+^ (right). *(B)* The effect of incremental addition of CaM WT (N−C) and of an equimolar mixture of the lobes (N&C) obtained in the absence of Ca^2+^ (left, 10 mM EGTA added) and in the presence of 100 µM free Ca^2+^ (right). (*C*) Comparison of the arithmetic addition of the curves obtained for each individual lobe (N+C) with the effect of an equimolar mixture (N&C) and with CaM (N−C) at concentrations under 200 nM in absence (left) or in the presence of 3.9 µM Ca^2+^ (that were indistinguishable from the results obtained in the presence of 100 µM Ca^2+^). *(D)* Plot of the reduction in fluorescence at the indicated concentration of competing lobe(s). ***, significance at P≤0.001, **P≤0.01, unpaired Student’s t test. *(E)* A model for the Ca^2+^-dependent CaM/SK2 interaction that can be derived from this set of experiments. 1. Both lobes cooperate in binding and the C-lobe, but not the N-lobe, is bound to SK2 in absence of Ca^2+^. 2. Ca^2+^ does not affect the interaction with the C-lobe. As the Ca^2+^ concentration increases the N-lobe becomes calcified. 3. The calcified N-lobe binds to SK2, leading to the observed increase in affinity in the presence of Ca^2+^. The data do not allow the oligomerization state to be established and therefore the dimerization of the CaM/SK2 complex that takes place upon Ca^2+^ binding is based on the resolved structure [9].

We examined the ability of the isolated N- and C-lobes to interact with SK2 CaMBD in the presence and absence of Ca^2+^ (Fig. 2 and Table 1). This assay exploits the fact that the isolated lobes of CaM are known to retain their native structure and that most properties of CaM are recapitulated by the summation of those of the lobes [7]. The interaction with the C-lobe was stronger than with the N-lobe, whereas the difference was reduced in the presence of Ca^2+^ because of the increased affinity to the N-lobe, while that of the C-lobe remained essentially the same (Fig. 2 *B*), consistent with structural and functional data (Fig. 2 *E*) [9]; [33]; [34].

**Table 1:** Summary of the binding parameters obtained after fitting a two sites Hill equation to the SK2 data in Fig. 2.

		Bmax_1_	IC50_1_ (nM)	h1	Bmax_2_	IC50_2_ (nM)	h2
**−Ca^2+^**	**N**	59.7±13.8	183±97	0.9±0.1	40.3±13.8	1875±187	2.9±1.1
	**C**	67.8±9.5	22.2±4.7	1.5±0.1	32.2±9.5	152±18.2	3.4±1.1
	**N&C**	93.3±0.8	16.1±1.8	1.4±0.2	6.7±0.8	189±13.1	≥5
	**N−C**	36.4±5.4	2.1±0.3	3.0±0.2	63.6±5.4	21.7±2.4	2.3±0.4
**+Ca^2+^**	**N**	30.1±7.5	16.3±3.4	3.3±1.4	69.9±7.5	131±36	1.5±0.3
	**C**	45.1±6.1	14.2±1.9	2.7±0.6	54.9±6.1	99.1±8.9	3.1±0.5
	**N&C**	28.2±2.7	11.6±0.6	≥5	71.8±2.7	15.9±3.1	1.6±0.3
	**N−C**	33.1±6.8	2.4±0.3	≥5	66.9±6.8	11.8±1.1	3.2±0.7

See legend to Fig. 2 for the experimental conditions used. h: Hill coefficient; IC_50_: concentration producing 50% inhibition.

The affinity of CaM, with both lobes linked together (denoted N−C, cyan line in Fig. 2), was higher than that of an equimolar mixture of the lobes (denoted N&C on the green line of Fig. 2 *A*). This difference was more evident at concentrations under 200 nM (Fig. 2 *C* and *D*). The effect of an equimolar mixture within this concentration range was larger than that produced by the arithmetic addition of the individual lobes (denoted N+C, purple line, Fig. 2 *C* and *D*), particularly at low lobe concentrations. These results mean that both lobes cooperate and that the linkage of the lobes favors binding of CaM to SK2.

### The Interaction with Q2AB is Dominated by the N-lobe in the Absence of Calcium and by the C-lobe in the Presence of this Ion

**Figure 3:**
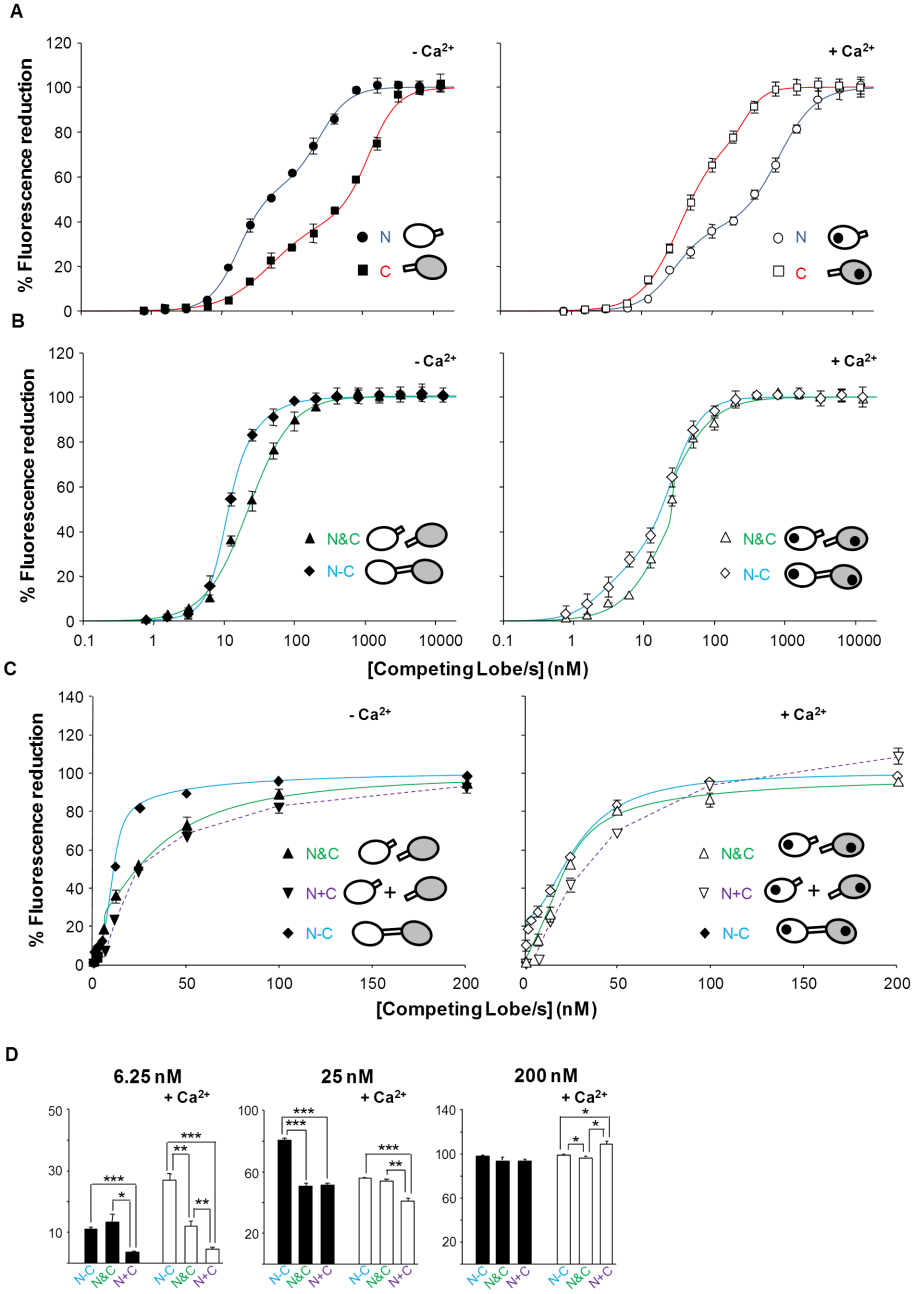
Q2AB binds preferentially to the N-lobe in the absence of Ca^2+^ and to the C-lobe in the presence of Ca^2+^. Relative concentration-dependent reduction in 12.5 nM D-CaM fluorescence emission when complexed with Q2AB. To achieve maximal sensitivity in the assay, concentrations of Q2AB that caused 50% of the maximal increase in D-CaM fluorescence emission were used (27 nM and 11 nM in the presence and absence of Ca^2+^, respectively). D-CaM was mixed with Q2AB and then each lobe (N or C), both lobes (N&C) or intact CaM (N−C) was added incrementally at the concentrations indicated. The data represent the means ± standard error from three or more independent experiments. Some error bars were smaller than the symbols. The result of fitting a two sites Hill equation to the competition curves is compiled in Table 2. *(A)* The effect of incremental addition of the indicated lobes obtained in the absence of Ca^2+^ (Left, 10 mM EGTA added) and in the presence of 100 µM free Ca^2+^ (right). *(B)* The effect of incremental addition of CaM WT (N−C) and of an equimolar mixture of the lobes (N&C) obtained in the absence of Ca^2+^ (left, 10 mM EGTA added) and in the presence of 100 µM free Ca^2+^ (right). (*C*) Comparison of the arithmetic addition of the curves obtained for each individual lobe (N+C) with the effect of an equimolar mixture (N&C) and of CaM (N−C) at concentrations under 200 nM in absence (left) or in the presence of 3.9 µM Ca^2+^ (that were indistinguishable from the results obtained in the presence of 100 µM Ca^2+^). *(D)* Plot of the reduction on fluorescence at the indicated concentration of competing lobe(s). ***, significance at P≤0.001, **P≤0.01, *P≤0.05, unpaired Student’s t test.

In the absence of free Ca^2+^, both lobes interacted with Kv7.2 (Fig. 3 *A*, red and blue lines), although unlike the interaction with SK2, the N-lobe displayed higher affinity than the C-lobe. In addition, the difference between an equimolar mixture (N&C) and the arithmetic addition of the effect of the lobes (N+C) at low concentrations was much smaller than that observed with SK2. By contrast, the affinity for CaM was higher than that of the equimolar mixture of the lobes (Fig. 3 *B* and *C*). Thus, like SK2, linkage of the lobes favors binding to Q2AB.

Calcium changed the interaction mode of both lobes, weakening binding to the N-lobe (Fig. 3 *A*, blue line) and strengthening the interaction with the C-lobe (Fig. 3 *A*, red line; see Table 2). The effect of holo-CaM (N−C) was again larger than that of the equimolar mixture of lobes (N&C), although such differences were no longer relevant at concentrations ≥25 nM. In addition, the effect of an equimolar mixture of lobes (N&C) at low concentration differed from that of the arithmetic addition of lobes (N+C) (Fig. 3 *C* and *D*), suggesting the existence of cooperativity.

**Table 2:** Summary of the binding parameters obtained after fitting a two sites Hill equation to the Q2AB data in Fig. 3.

		**Bmax_1_**	**IC50_1_ (nM)**	**h1**	**Bmax_2_**	**IC50_2_ (nM)**	**h2**
**−Ca^2+^**	**N**	55.3±4.1	17.3±1.6	2.1±0.2	44.7±4.1	240±28	2.0±0.2
	**C**	41.6±6.3	49.7±11.1	1.2±0.2	58.4±6.3	1224±133	2.1±0.3
	**N&C**	10.3±4.6	6.1±2.8	≥5	89.7±4.6	25.1±1.4	1.4±0.1
	**N−C**	62.5±7.1	10.3±0.5	3.4±0.6	37.5±7.1	18.6±5.2	1.5±0.2
**+Ca^2+^**	**N**	37.2±2.9	28.9±3.4	1.8±0.2	62.8±2.9	882±69	1.6±0.1
	**C**	77.6±4.5	35.7±3.2	1.5±0.1	22.4±4.5	303±33.1	3.1±0.7
	**N&C**	82.8±2.1	21.5±2.1	1.3±0.1	17.2±2.1	22.1±2.6	≥5
	**N−C**	25.9±8.1	2.6±0.9	1.7±0.5	74.1±8.1	24.4±2.5	1.9±0.2

See legend to Fig. 3 for the experimental conditions used. h: Hill coefficient; IC_50_: concentration producing 50% inhibition.

### The CaM Interaction with Q2AB is Dominated by Helix B in the Absence of Calcium and by Helix A in the Presence of this Ion

**Figure 4:**
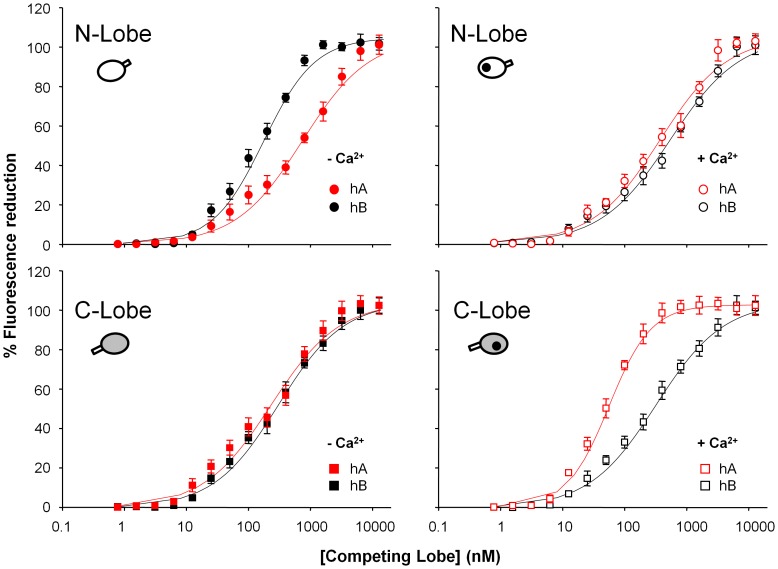
The N-lobe binds preferentially to helix B in the absence of Ca^2+^, whereas the C-lobe binds preferentially to helix A in the presence of Ca^2+^. Competition curves with isolated CaM lobes (N or C) obtained using the individual CaM lobes and performed in the absence (filled symbols) or in the presence of Ca^2+^ (open symbols). D-CaM (12.5 nM) was mixed with helix A (hA, red symbols) or helix B (hB, black symbols) at a concentration corresponding to its calculated EC_50_ for the increase in D-CaM fluorescence emission (46.4 and 65.6 nM in absence or presence of Ca^2+^ for helix A respectively, and 20.1 and 42.6 nM in absence or presence of Ca^2+^ for helix B respectively) and then each lobe was added incrementally at the concentrations indicated. The data represent the means ± standard error from three or more independent experiments, where some error bars were smaller than the symbols. The result of fitting a Hill equation to the competition curves is compiled in Table 3.

The contribution of each individual segment of the Kv7.2 binding site was examined using the competition assay (Fig. 4). Although the binding of CaM with Kv7.2 is not fully recapitulated by its individual segments [25], this analysis helps in understanding how CaM interacts with the channel.

In the absence of Ca^2+^ the interaction with the N-lobe is dominant (Fig. 3 *A*), and the affinity of this lobe is higher for helix B (Fig. 4 and Table 3). The affinities of the C-lobe for either helix were essentially the same, albeit lower than the affinity of the N-lobe for helix B. By contrast, in the presence of Ca^2+^ when the interaction with the C-lobe dominates (Fig. 3 *A*), the interaction of highest affinity was that between the C-lobe and helix A. Overall, these data revealed that Ca^2+^ strengthened the interaction of helix A with both lobes, whereas for helix B a reduction in the affinity of the interaction with the N lobe was observed.

**Table 3:** Summary of the binding parameters obtained after fitting a one site Hill equation to the data in Fig. 4.

			**IC_50_ (nM)**	**h**	**Kd (nM)**
**−Ca^2+^**	**hA**	**N**	533±51	0.9±0.1	530±55
		**C**	181±21	0.8±0.1	169±23
	**hB**	**N**	130±8	1.1±0.1	119±9
		**C**	238±16	0.9±0.1	231±18
**+Ca^2+^**	**hA**	**N**	284±33	0.9±0.1	267±37
		**C**	46±2	1.4±0.1	17±2
	**hB**	**N**	415±42	0.8±0.1	414±48
		**C**	244±17	0.9±0.1	229±21

See legend to Fig. 4 for the experimental conditions used. h: Hill coefficient; IC_50_: concentration producing 50% inhibition. Kd: affinity constant derived assuming a 1∶1 binding.

### NMR Reveals Interactions of Q2AB with the N-lobe in Absence of Ca^2+^ and with Both Lobes in the Presence of this Ion

**Figure 5:**
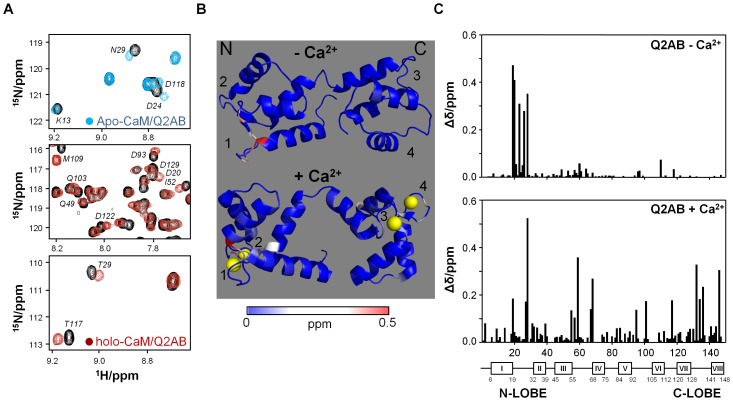
Monitoring of the interaction between CaM and Q2AB by (^1^H, ^15^N)-HSQC spectroscopy. *(A)* Details of ^1^H,^15^N-HSQC spectra of CaM in the absence (black) and in the presence of 2.5 equivalents of Q2AB for apo-CaM (blue) and holo-CaM (red). *(B)* Structural mapping of the chemical shift perturbation (CSP) induced by Q2AB binding to apo-CaM (top, PDB entry 1CFC) and holo-CaM (bottom, PDB entry 2K0E). The lobes are indicated by the labels “N” and “C”, and the four EF hands are numbered 1–4. The CSPs are color coded as indicated by the gradient bar. Figure created using Pymol. *(C)* CSPs induced by Q2AB to apo-CaM (top) and holo-CaM (bottom) plotted in function of the CaM residue number.

NMR data confirmed that Ca^2+^ affects the interaction of Q2AB with both lobes of CaM as evident in the chemical shift perturbations (CSP) produced by Q2AB in the ^1^H-^15^N-HSQC map for apo-CaM and holo-CaM (Fig. 5 and Fig. S6 in File S1). In response to Ca^2+^ the protein undergoes a significant conformational rearrangement and, consequently the HSQC spectra for apo-CaM and holo-CaM differ significantly. Upon Q2AB binding, some CaM resonances in the spectrum were perturbed (Fig. 5 *A*), although the overall spectrum is similar to that of the corresponding peptide free proteins. The CSPs are represented in the structure of CaM in Fig. 5 *B* and quantitatively shown in Fig. 5 *C*. Such perturbations in the holo-CaM map are consistent with a model where Q2AB make contact with the two lobes. Interestingly, when apo-CaM is monitored, the residues that belong to the N-lobe almost exclusively show altered chemical shifts, consistent with the dominant effect of the N-lobe in the absence of Ca^2+^ evident in the competition assay (Fig. 3 *A*, blue line, see Fig. S7 in File S1).

In the absence of Ca^2+^ all the residues that show significant changes in the NMR spectrum are located in the first loop that coordinates Ca^2+^ binding in EF1. The most important changes in apo-CaM’s chemical shift (with changes higher than 0.25 ppm) corresponded to the residues F19, D20, G23, T26 and T28. Thus, two residues directly involved in Ca^2+^ coordination, D20 and T26, also undergo significant CSPs upon binding to Q2AB (0.41 and 0.28 ppm, respectively). Upon CaM calcification, remarkable differences were found in the CSP map of the EF1 moiety. Notably, F19 and T28, were two residues significantly affected by the interaction with the Q2AB both in the presence (0.18 and 0.50 ppm, respectively) and absence of Ca^2+^ (0.47 and 0.35 ppm, respectively). These residues are located in EF1 and they are not directly involved in Ca^2+^ coordination. A signal for I27 was also evident, whereas the remaining EF1 residues were unaffected in the presence of Ca^2+^.

For holo-CaM, residues belonging to the two domains demonstrated CSPs. In addition to the residues described in EF1, significant CSPs were noted in I27 of EF1, and in G59 and F68 of EF2. In the C-lobe, CSPs in EF3 (D95 and S101) and EF4 (G132, G134 and V136) were also observed. At the junction between EF3 and EF4, T117 also presented CSPs, as did T146 at the C terminus. Interestingly, G132, G134 and V136 are in the loop in EF4 that participate in Ca^2+^ coordination within the C-lobe, suggesting that Q2AB might influence Ca^2+^ binding.

**Figure 6:**
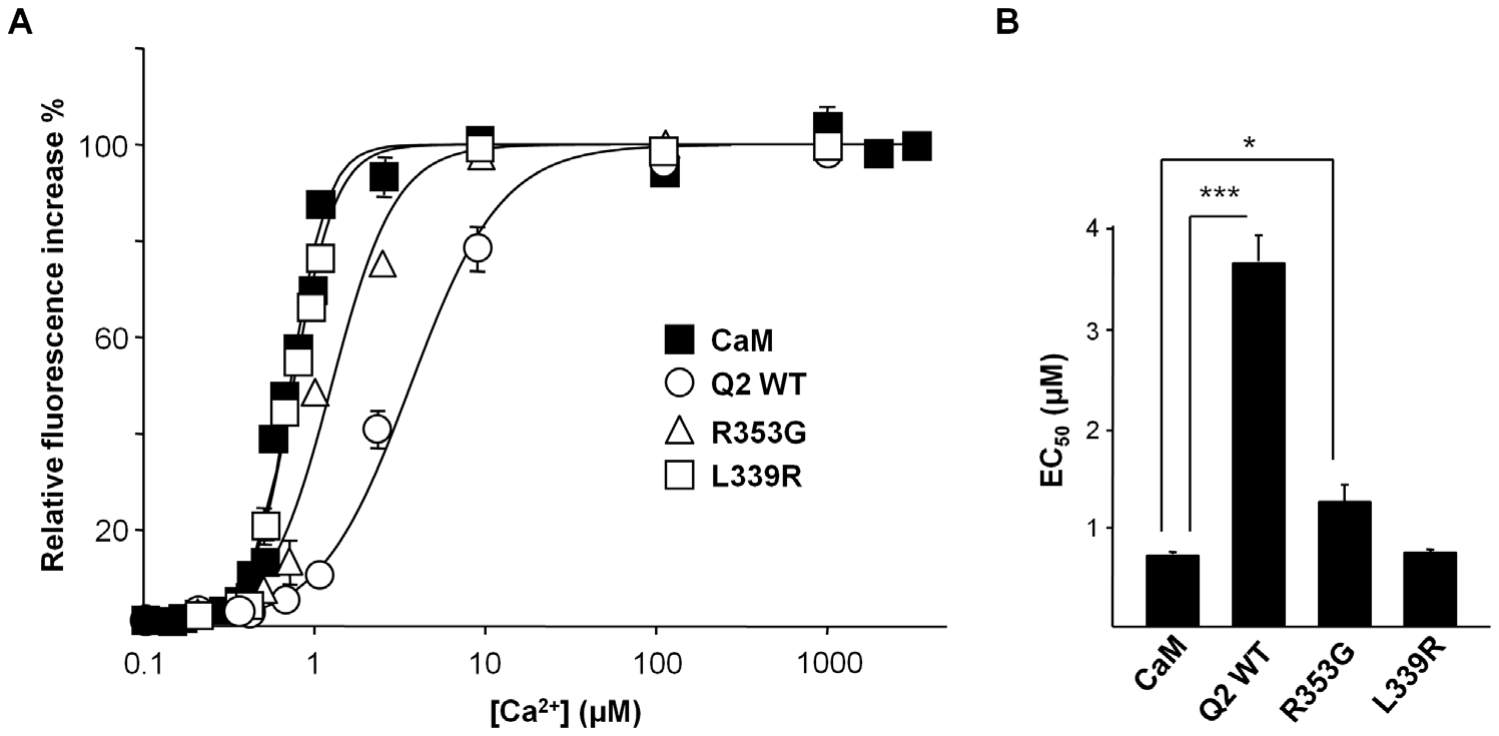
Q2AB weakens the Ca^2+^-CaM interaction. *(A)* Relative increase in D-CaM fluorescence emission (12.5 nM) in response to increased Ca^2+^ concentrations in the presence (open circles) or absence (filled circles) of a molar excess of Q2AB (200 nM) or the indicated segment A mutants. We have previously shown that maximal D-CaM fluorescence is attained at this concentration for WT, L339R and R353G [26]. The lines are the result of fitting a Hill equation to the data. The data represent the means ± standard error from three or more independent experiments. Some error bars were smaller than the symbols. The EC_50_ values obtained are (in µM): CaM = 0.72±0.02, CaM/Q2AB WT = 3.64±0.26, CaM/Q2AB R353G = 1.26±0.16, CaM/Q2AB L339R = 0.75±0.02. *(B)* Plot of the apparent binding affinity derived from the data in A. ***, significance at P≤0.001, *P≤0.05, unpaired Student’s t test.

Thus, the NMR data show that the CaM Ca^2+^-binding sites 1 and 4 are affected by this interaction, suggesting that the affinity for this ion may be altered in the complex. We examined the Ca^2+^ binding affinity in the presence of Q2AB, which shifted from 0.7 for CaM to 3.6 µM in the CaM/Q2AB complex, respectively (Fig. 6). However, this analysis does not distinguish between individual sites, and the contribution of each EF hand to the change in the Ca^2+^ binding affinities is unknown.

Protein aggregation of the individual helices occurred at the concentration required for NMR, precluding a full study. Nevertheless, we were able to obtain data from a peptide derived from segment A (pA) in the presence of Ca^2+^. The CSP map was similar to that obtained for Q2AB (see Supporting Material) and as such, helix A can interact with both the N- and C-lobes of holo-CaM in the absence of helix B. We also used NMR spectroscopy to explore the impact of mutations in helix A (I340E and A343D) and helix B (S511D) that impair CaM binding [10]; [17]; [26], and indeed we found no evidence of binding. However, we did observe CSPs in Ca^2+^ bound CaM when testing the two helix A BFNC-causing mutants that partially disrupt CaM binding (L339R and R353G [26]). Partial assignment of the mutants was achieved by chemical shift comparison with the wild type protein. For the dataset available, the CSP maps exhibited a similar profile for both mutants and the mutations provoked a diminution in the CSP, reflecting the reduction in the strength of binding. Most of the perturbations mapped to the holo-C-lobe, consistent with the predominant interaction of this lobe identified in the competition assays. Compared to the WT, the signals from the residues in EF1 were absent in the two mutants (see Fig. S8 in File S1). These results suggest a major role of EF1 and EF4 in the transduction of the signal for Kv7.2. The affinity for Ca^2+^ in the CaM/Q2AB complex was reduced in the R353G mutant-CaM complex but not as much as in the WT-CaM complex (from 0.7 to 1.3 µM *vs* to 3.6 µM, respectively), whereas no significant changes were observed in the complex with the L339R mutant (Fig. 6). Thus, the changes in affinity paralleled the changes in CSP.

## Discussion

In general, the interaction of CaM with ion channels and other targets is dominated by the C-lobe [33]–[38]. We show here that Kv7.2 does not conform to this rule but rather the N-lobe plays the more dominant role in apo conditions. This conclusion was reached on the basis that most of the disturbances on CaM CSPs take place at EF1 in the N-lobe, and the results of competition assays. Furthermore, the data suggest that helix B is the main site of interaction with the apo-N-lobe, in agreement with direct binding analysis [25].

The data reveals significant differences in the binding strength of the CaM components that are especially evident at low concentrations. The concentration of free apo-CaM within the cell is uncertain, with reported values ranging from low (60 nM) to high (8800 nM) [39]; [40]. Functional analysis of Kv7.2 channels favors the idea that free apo-CaM is limiting [25]. At low concentrations, our data reveals that the binding of the lobes is not independent but that cooperativity occurs between the lobes, especially when binding to SK2. In addition, the fact that the two lobes are linked together also favors their binding to both SK2 and Q2AB. When one lobe of CaM binds to the channel, the other lobe will be positioned in the proximity of the binding site, thereby augmenting its effective concentration and enhancing its interactions. In addition, the linker itself may contribute to binding, as shown for SK2 channels [41]. A differential contribution of the linker may explain the larger difference between binding to CaM and to an equimolar mixture of lobes observed with SK2 than with Q2AB (compare the data at 6.25 nM of Figures 2 *B* and 3 *B*). Other factors, such as the binding to the one lobe sterically impeding the association with the linker, may contribute to this difference.

**Figure 7:**
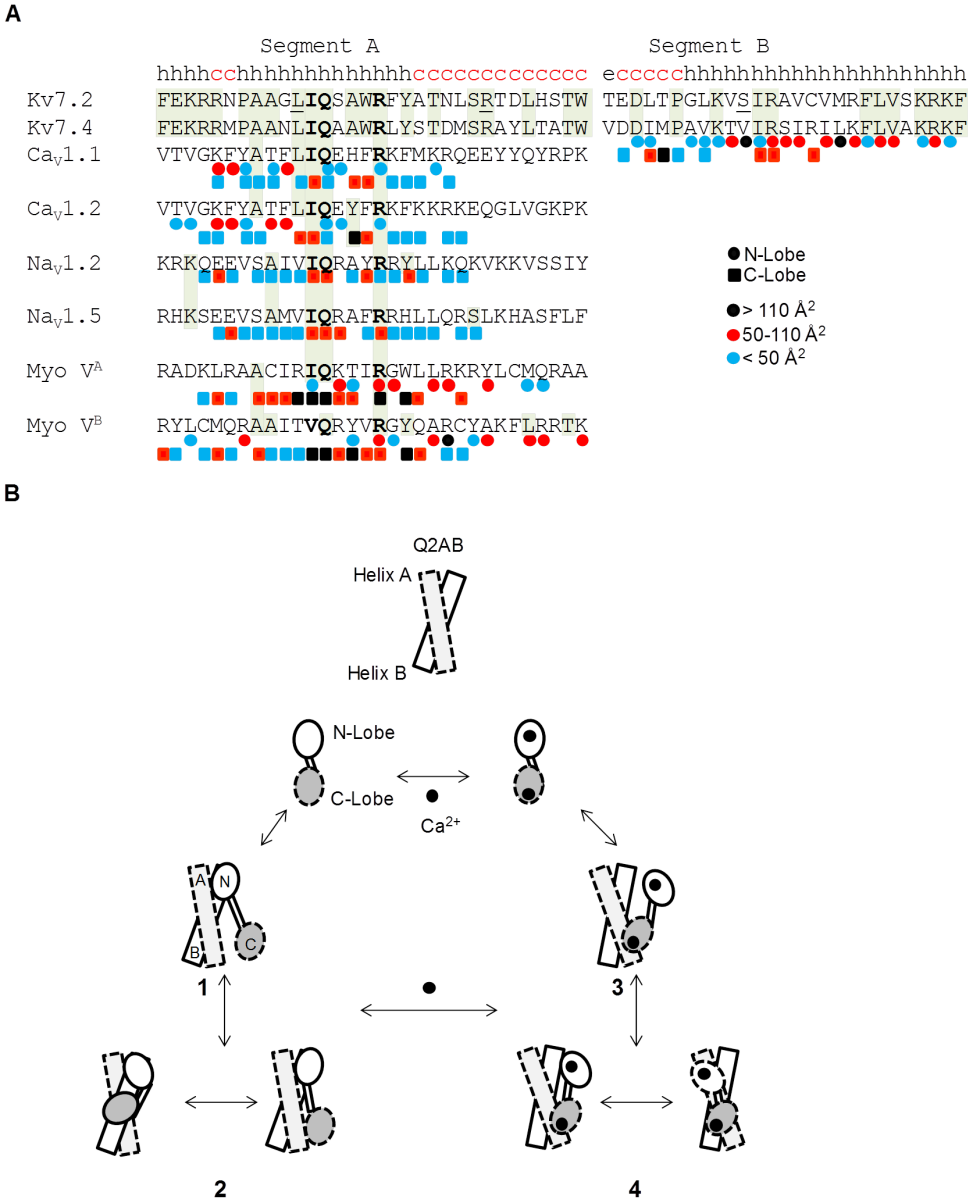
Model for the Ca^2+^-dependent CaM/Q2AB interaction. *(A)* Sequence alignment of segments A and B in which L339, R353 and S511 are underlined. The predicted secondary structure of Kv7.2 according to the GORV algorithm is indicated above the sequence (http://gor.bb.iastate.edu/, h = alpha helix, e = extended, c = coiled). The circle beneath a residue indicates that it contacts the N-lobe, while those in contact with the C-lobe are indicated with a square, both of which are color coded according to the CaM surface contact. The contact surface area has been estimated using the Sobolev *et al.* algorithm [52]. *(B)* Interaction model. The Q2AB helices are depicted as rectangles, the CaM lobes as ovals. Binding to CaM is transient [23]; [24], and the interaction with helix A is critical for function [25]. Given the greater affinity for helix B [22]; [25], it is more likely that CaM docks initially to this helix *via* the N-lobe, facilitating the interactions between the C-lobe and helix A. Subsequently, a dynamic equilibrium is established: 1.- In the absence of Ca^2+^ the N-lobe dominates the interaction and initially binds to helix B. 2.- Subsequently, the C-lobe engages, establishing an equilibrium between binding to helix A and helix B. 3.- In the presence of Ca^2+^ the C-lobe binds to the IQ site of helix A. 4.- The holo-N-lobe alternates between helix A and helix B. Upon calcification, the interaction between helix B and the N lobe is weakened and the binding between helix A and the C-lobe becomes more significant. Concomitantly, the global affinity in the presence of Ca^2+^ is reduced.

NMR spectral changes *per se* do not readily identify the residues interacting with a target, although we noted that in some of the structures deposited in the PDB, the Ca^2+^-loaded CaM residues not involved in Ca^2+^ coordination that correlated with the CSPs from the two Kv7.2 mutant analyzed (F19, E115, A128, Q143) made contacts with the target. These CaM residues also contribute to binding to Ca_V_1.2 [28]; [37]; [42], together with V136 and other residues also identified in the map of Q2AB (E14, F19, L32, V55, F68, M144 and T146), although T117 does not make contacts with the target. All these residues, except V55, contact SK2b [6] and DAP Kinase B [43]. With the exception of V136, both calcineurin and myosin light chain kinase (MLCK) contact all these residues [44]; [45]. The holo-C-lobe of these structures adopts a similar configuration, although the targets assume different angles. All the targets are located in a plane that is almost perpendicular to the first helix of the EF3 hand (helix VI), and they converge onto a point close to where the IQ residues of Ca_V_1.2 are located. Helix A of Kv7 contains an IQ binding motif similar to that found on Ca_V_1.2. The IQ site can be divided into three components: pre-IQ, IQ-core, and post-IQ. The IQ-core ([I,L,V]QxxxR) is highly conserved in many CaM targets [46]. Based on mutagenesis and on the existing structural data (see Fig. 7 *A*), it has been proposed that the C-lobe docks onto the IQ-core while the N-lobe may remain free, or alternatively it may dock at the pre-IQ or the post-IQ sites [47]. The structure of the IQ motif interacting with apo-CaM has been elucidated for Na_V_1.5, and no interactions with the apo-N-lobe were evident [38]. In addition, a complex of the individual apo-C-lobe with Na_V_1.2 has been resolved [36], emphasizing that the interaction of the IQ site with the apo-C-lobe is autonomous. Based on the invariance of the apo-C-lobe/protein complexes and the sequence homology of Kv7.2 segment A with the IQ sites of myosin V, neurogranin, Na_V_1.2, Na_V_1.5, Ca_V_1.1 and Ca_V_1.2, we expect that helix A adopts a similar orientation, leaving the N-lobe free to interact with the pre- or post-IQ sites or with helix B.

A distinction must be made between productive and transient interactions. By productive interactions we refer to those that can be detected directly by common biochemical techniques, as opposed to transient interactions that are difficult to detect. Both CaM lobes present non-productive interactions with the isolated helix A and with the isolated helix B [10]; [25], predicting a transient stoichiometry of 1∶2. For the productive complex, we favor the possibility of the C-lobe interacting with the IQ site in helix A and the N-lobe interacting with helix B, such that the resulting complex would comply with the detected 1∶1 stoichiometry [21]; [22]. These contacts set the stage to establish a productive interaction with the full CaMBD, consistent with our earlier observation showing that both helices A and B are required for stable binding to apo-CaM [10]; [25].

In the presence of Ca^2+^ there is a clear increase in the strength of SK2 binding to the N-lobe, which is consistent with functional and structural data [9]; [33]. By contrast, while the interaction of Q2AB with the C-lobe is enhanced in such conditions, the interaction with the N-lobe is weakened. The analysis with the isolated helices shows that in the presence of Ca^2+^ helix A has a significantly higher affinity for the C-lobe than helix B (see Table 3). In contrast, the affinities of the holo-N lobe for helix A or helix B are comparable. Recently, the structure of helix B of Kv7.4 complexed with holo-CaM (4GOW) was resolved [22], revealing that segment B adopts an alpha helical configuration and that it makes extensive contacts with the N-lobe. Although segment A is missing, it is expected that this structure will share features with the full Kv7 CaMBD/CaM complex. Satisfactorily, most of the holo-N-lobe residues outside the Ca^2+^ binding loops that were identified on the displacement map when complexed with Q2AB (E14, F19, L32, L39, V55 and F68) contact segment B of Kv7.4 (see Fig. 7 *A*). The picture is different for the C-lobe, as only V91, M144 and, to a lesser extent, F141 make contact with the initial portion of helix B in Kv7.4. Notably, K115, T117, A128, Q143 and T146 do not interact with Kv7.4. By contrast, almost every C-lobe residue pinpointed in our CSP analysis contacts the IQ site of Ca_V_1.2 (holo form, with the exception of T117) or Na_V_1.5 (apo form, with the exception of Q143). This discrepancy may be explained by helix A preventing the interaction between the C-lobe and the initial portion of segment B in the complete CaMBD, instead promoting an interaction between the C-lobe and segment A. Since helix A is missing in the solved Kv7.4 complex, the C-lobe is free to interact with pre-segment B, leading to this marked difference. The resolved CaM/Kv7.4-segment-B structure may therefore correspond to an intermediary in the formation of a productive complex with the complete Kv7 CaMBD.

A remarkable relationship exists between the *in vitro* D-CaM fluorescent changes and the impact of helix A BFNC-causing mutations on M-current density [26]. The present data extend this relationship to the impact of those mutations on the CSPs, as well as to the impact on the Ca^2+^ binding properties of the complexes. Mutations in both helices A and B have been linked to KCNQ2-related epilepsies, with a wide range of phenotypic manifestations [48]; [49]. Although a systematic evaluation of the effect of all these mutations on CaM binding has not been performed, it is tempting to speculate that the clinical outcome might be related to the site of CaM/Q2AB interaction. For instance, mutations located at the pre-IQ site or helix B are associated with poorer clinical outcome than those located at the IQ and post-IQ sites [50]. Thus, understanding the relationship between the individual lobe/helix engagement and clinical severity may represent an important advance for the prognosis and management of KCNQ2-related pathologies.

A combination of structural analysis and molecular dynamics suggest that the CaM lobes are exploring an extensive landscape of conformations. In the absence of ligand, each lobe transiently adopts a conformation that matches that found when they are engaged with the target, and it is thought that a seeding complex with one lobe is formed by a lock and key mechanism [51]. At this point, the landscape of configurations of the other lobe is restricted, increasing the likelihood that this second lobe is found in a conformation complementary to its target and that it will become engaged by a lock and key mechanism. This priming step may be the basis of the cooperation between the lobes. Thus, the formation of the final complex follows a defined trajectory through a sequence of partial or local tridimensional engagements. Strong evidence for a coupled equilibrium shift has been provided for the interaction with MLCK in the presence of Ca^2+^, in which the ligand binds first to the C-lobe, and this facilitates the downstream binding to the N-lobe [51]. Within the frame of the coupled equilibrium shift theory [51], our results are consistent with the hypothesis that in the presence of Ca^2+^ the IQ site of helix A docks preferentially to the C-lobe of holo-CaM paving the way for binding to the N-lobe (Fig. 7 *B*). The N-lobe could at this stage engage either with helix B or helix A. The proximity and the relative affinities of the N-lobe/helix complexes suggest that the holo-N-lobe will tend to engage more often with helix A, although structural constrains may shift this balance. In turn, these interactions are coupled to the reaccomodation of some Ca^2+^ binding loops (in particular EF1 and EF4), resulting in a reduced affinity of CaM for Ca^2+^. In the absence of Ca^2+^, helix B docks preferentially to the N-lobe of apo-CaM paving the way for binding with the C-lobe (Fig. 7 *B*). The C-lobe could at this point bind either to helix B or helix A (the difference between the apparent affinities for the apo-C-lobe/helix A and apo-C-lobe/helix B complexes is relatively small, see Table 3). However, structural constrains, such as a postulated kink before helix B (see Fig. 7 *A*), may preferentially direct the apo-C-lobe to the IQ site in helix A. Based on the properties of the isolated components, other binding modes and different stoichiometries are likely to exist transiently. Thus, we envisage a dynamic CaM/Q2AB complex, in which the lobes engage alternatively with different helices. Thus, the simultaneous positive/negative lobe cooperativity is a substrate for a very dynamic behavior of the CaM/Kv7 complex, and we can imagine CaM as a cradle holding Kv7.2, and Ca^2+^ as the hand that rocks the cradle.

## Supporting Information

File S1 **Supporting Material and Figures S1–S8.**
(PDF)
