# Supplementary material for: Pivoting between Calmodulin Lobes Triggered by Calcium in the Kv7.2/Calmodulin Complex
Source: PLoS One. 2014 Jan 28;9(1):e86711. doi: 10.1371/journal.pone.0086711 (PMC3904923; doi:10.1371/journal.pone.0086711)
Supplement: File S1 — Supporting Material and Figures S1–S8. (PDF) [file pone.0086711.s001.pdf]

SUPPORTING MATERIAL
for
PIVOTING BETWEEN CALMODULIN LOBES TRIGGERED BY CALCIUM IN THE Kv7.2/CALMODULIN COMPLEX

Alessandro Alaimo[†], Araitz Alberdi[†], Carolina Gomis-Perez[†], Juncal Fernández-Orth[†], Ganeko Bernardo-Seisdedos[†], Covadonga Malo[†], Oscar Millet[‡], Pilar Areso[§] and Alvaro Villarroel[†]*

[†]Unidad de Biofísica, CSIC, UPV/EHU, [§]Dept. Farmacología, UPV/EHU, Universidad del País Vasco, Barrio Sarriena s/n, 48940 Leioa, Spain; [‡]Structural Biology Unit, CICbioGUNE, Bizkaia Technology Park, Building 800, 48160 Derio, Spain.

***Correspondence: alvaro.villarroel@csic.es**

Short (Page Heading) Title: CaM/Kv7.2 binding

SDS-PAGE and Gel filtration. Recombinant proteins were checked for purity by Coomassie brilliant blue staining of SDS-PAGE employing 10 or 15% gels. Gel filtration was performed on a Superdex 200 HR 10/30 column on an AKTA FPLC (GE Healthcare) generally in Tris-HCl 20 mM, NaCl 100 mM, pH 7.5. The column was calibrated with molecular mass standards (Biorad), that included thyroglobulin (670 kDa), γ-globulin (158 kDa), ovalbumin (44 kDa), myoglobin (17 kDa) and vitamin B_{12} (1.35 kDa).

Dynamic light scattering. The oligomerization state of the purified proteins was then examined by dynamic light scattering (DLS) using a Zetasizer Nano instrument (Malvern Instruments Ltd.). Samples were passed through 0.22-μm membrane filters (Millipore) and centrifuged at 13.000 rpm for 10 min to clarify them from dust. Samples placed into single use plastic cuvettes were maintained at a fix temperature of 25 °C. The concentration of the proteins was 1-2 mg/ml and, generally, the buffer used was Tris-HCl 20 mM, NaCl 100 mM, pH 7.5. Measurements were made at an angle $\theta = 90°$ to the incident beam, and the data were collected at every 60 s. The correlation functions were analyzed to obtain the distributions of the decay rates and, hence, the apparent diffusion coefficients and ultimately the distributions of the hydrodynamic radius of the scattering particles in solution via Stokes-Einstein equation. Finally, the monodispersity or polydispersity of the solutions was assessed, and the molecular weights of the predominant species were calculated.

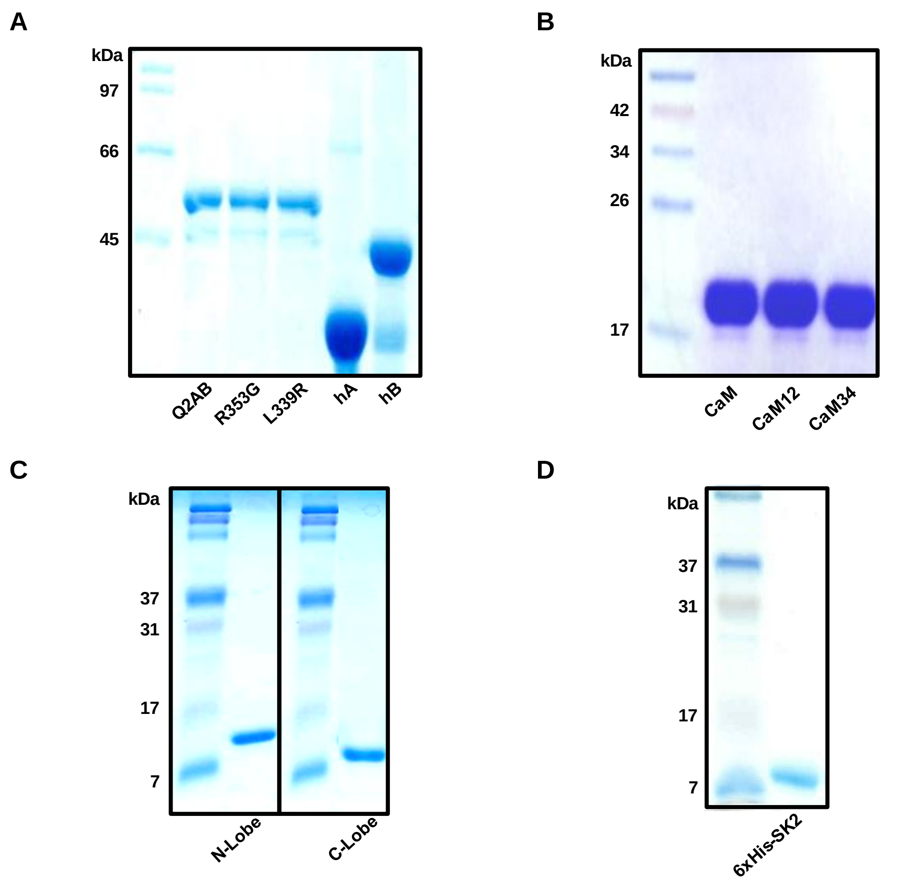

Fig. S1

Figure S1. SDS-PAGE analysis of the proteins employed in this study. Numbers denote molecular weight markers. *(A)* Kv7.2 recombinant proteins (10% gel). *(B)* Calmodulin and calmodulin mutants (15% gel). *(C)* Individual calmodulin lobes (15% gel). *(D)* SK2 calmodulin binding domain (15% gel).

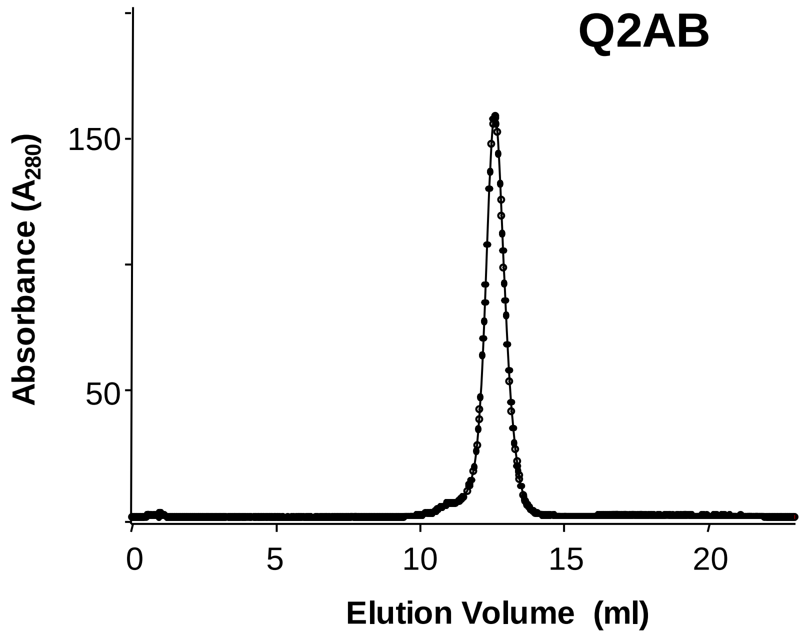

Figure S2. Size exclusion chromatography of the Q2AB performed using a Sephadex 200 HR 10/30 column in Tris-HCl 20 mM, NaCl 100 (pH 7.5).

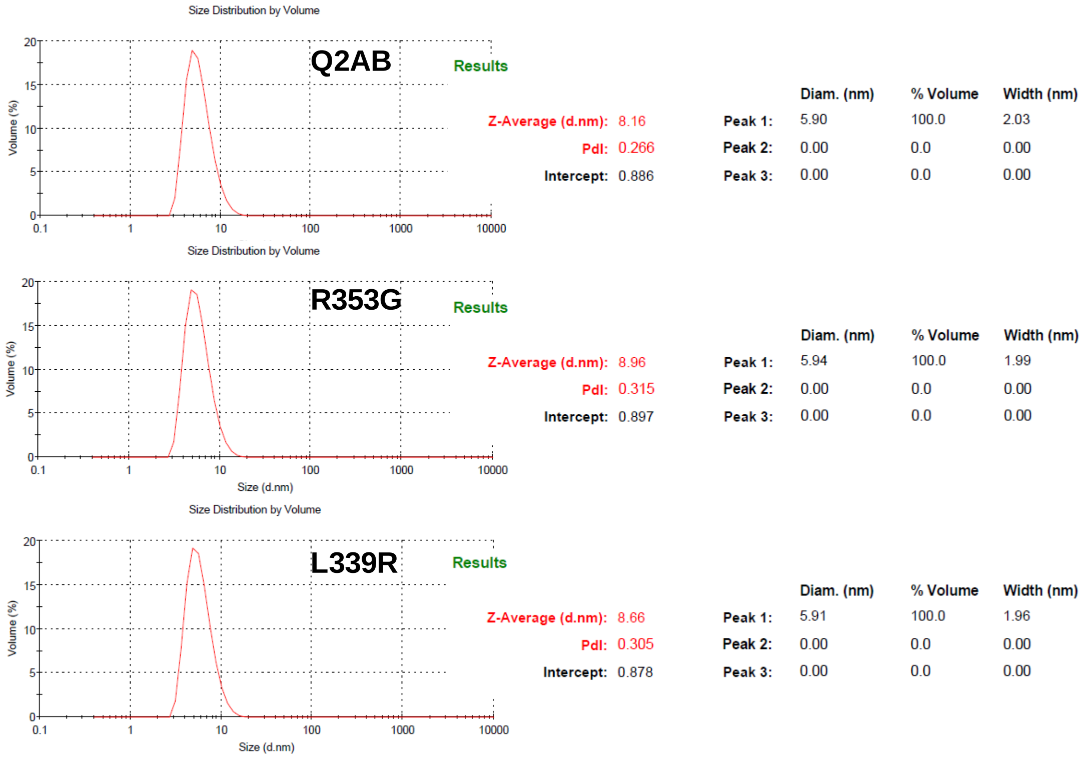

Figure S3. Dynamic light scattering (DLS) of Q2AB, R353G and L339R mutants. Representative volume-weighted size distribution of 1 mg/ml of the indicated proteins. Buffer conditions were Tris-HCl 20 mM, NaCl 100 (pH 7.5). Low value of Polydispersity Index (PdI), estimated from the apparent diameters assuming globular protein shape, and the presence of a unique peak exclude the presence of aggregate in these samples.

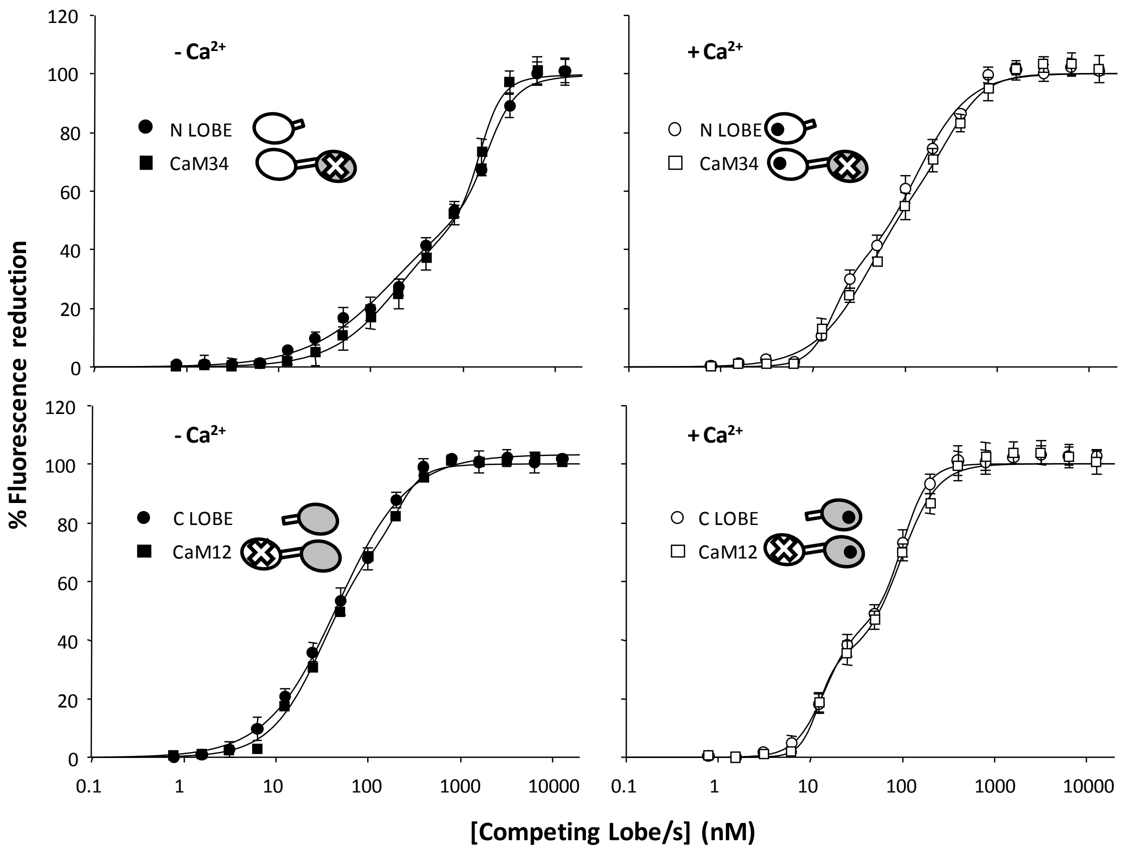

Figure S4. Competition curves with isolated CaM lobes (N or C) are compared with competition curves obtained with mutated CaM (CaM12 and CaM34). D-CaM (12.5 nM) was mixed with SK2 at a concentration corresponding to its calculated EC_{50} for D-CaM fluorescence emission increase (9.2 and 13.7 nM in presence or absence of Ca^{2+}, respectively) and the competing peptides were added incrementally at the concentrations indicated. The data represent the means ± standard error from three or more independent experiments.

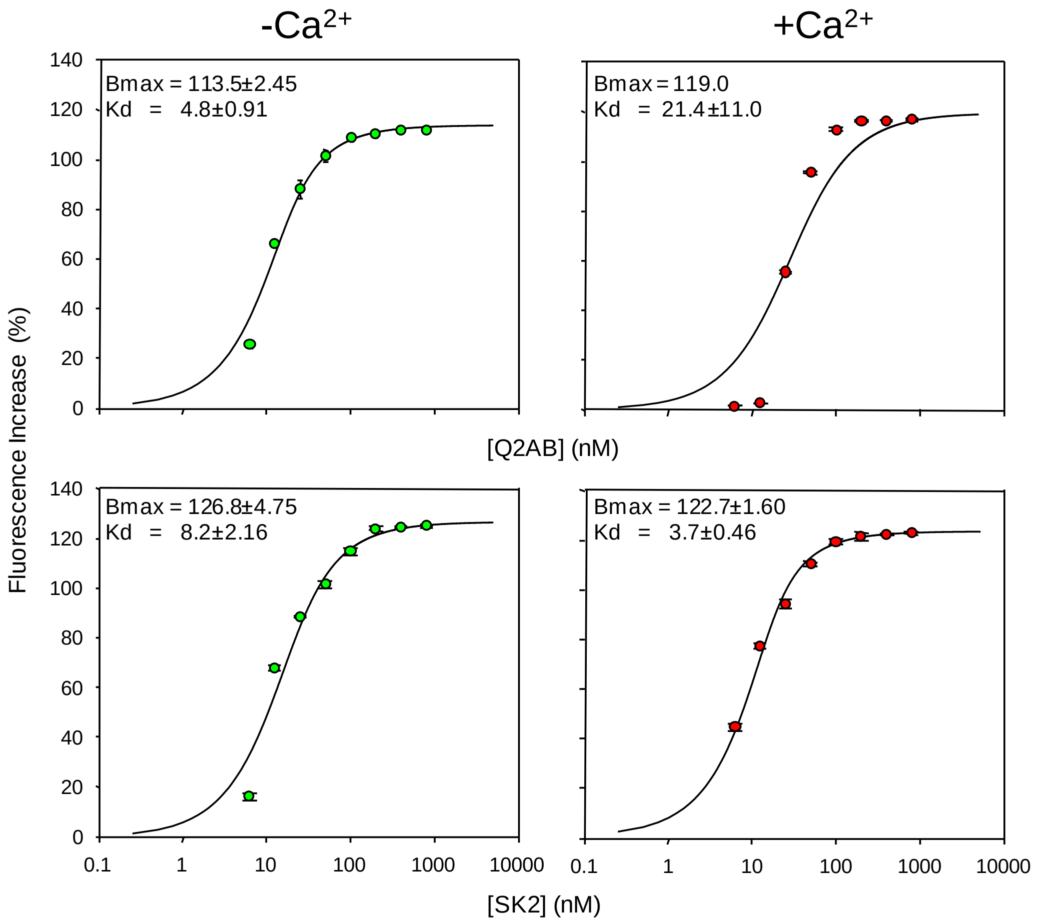

Figure S5.

The following equation, which assumes as 1:1 stoichiometry, was used to estimate the K_d from the data of Fig. S5 *B*:

$$F = F_{max} \times \left(1 - \frac{2 \times K_d}{([peptide] - [CaM] + K_d) + \sqrt{([peptide] - [CaM] - K_d)^2 + 4 \times K_d \times [peptide]}}\right.$$

Where F is the increase in fluorescence, F_{max} is a free parameter that represents the maximal fluorescence, [peptide] is the known concentration of total peptide, [CaM] is the known concentration of total D-CaM, and K_d is a free parameter for the affinity constant.

Continuous lines are the result of the Fit of the relative concentration-dependent enhancement of 12.5 nM D-CaM fluorescence emission by Q2AB (top panels) and SK2 (bottom panels) in the absence (left panels) and in the presence of 3.9 µM Ca^{2+} (right panels). Bmax was constrained to be ≤ 119 when fitting the equation to the Q2AB data in the presence of Ca^{2+}. The fit to the data in the presence of Ca^{2+} for Q2AB was very poor, suggesting that the CaM/Q2AB deviates from a 1:1 stoichiometry in the presence of Ca^{2+}.

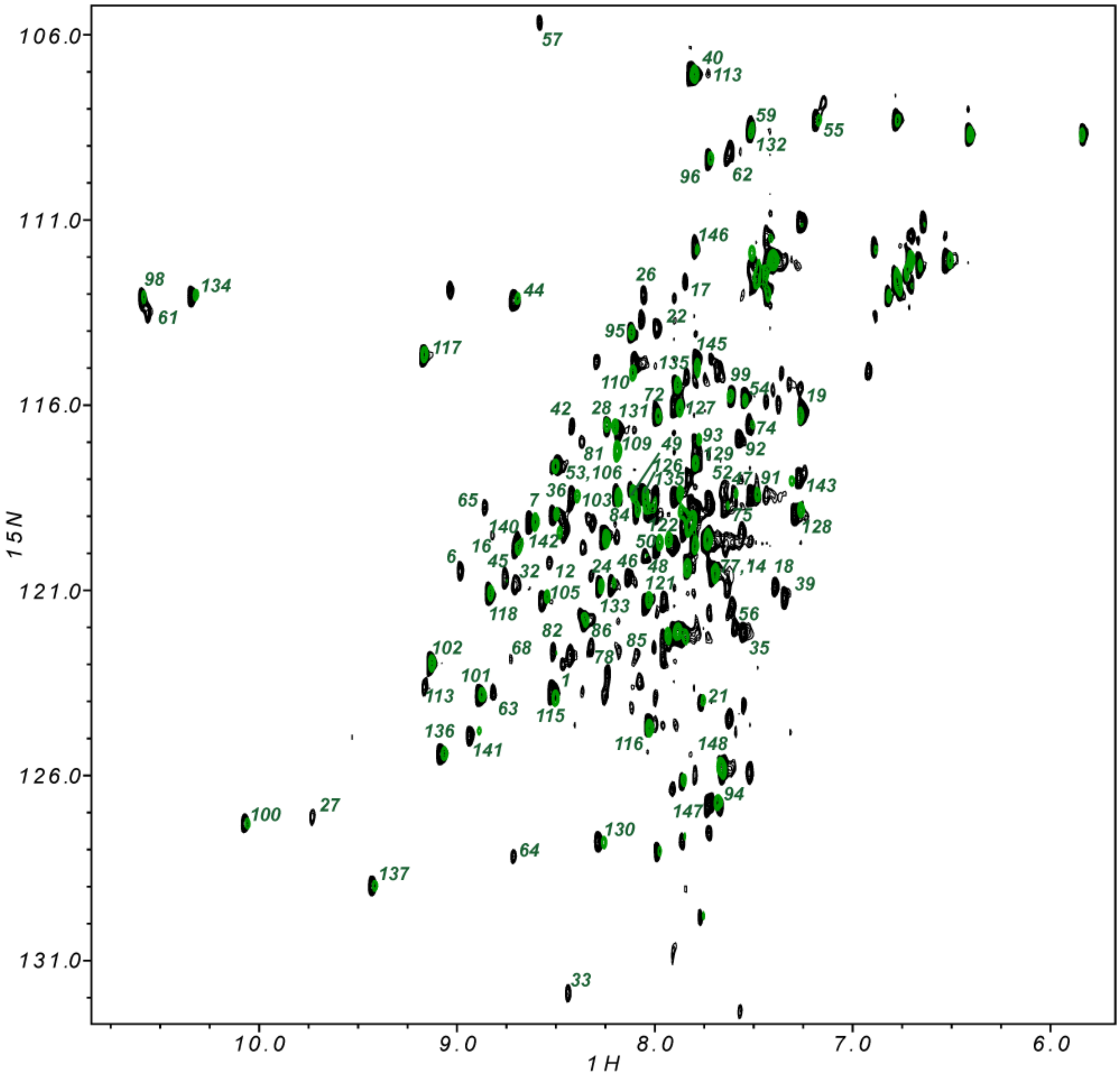

Figure S6. ^{1}H-^{15}N-HSQC spectrum of calcium bound calmodulin in the absence (black) and in the presence (green) of Q2AB peptide. Spectra are shown with equivalent S/N for comparison. Green numbers correspond to the residues of the complex that could be identified.

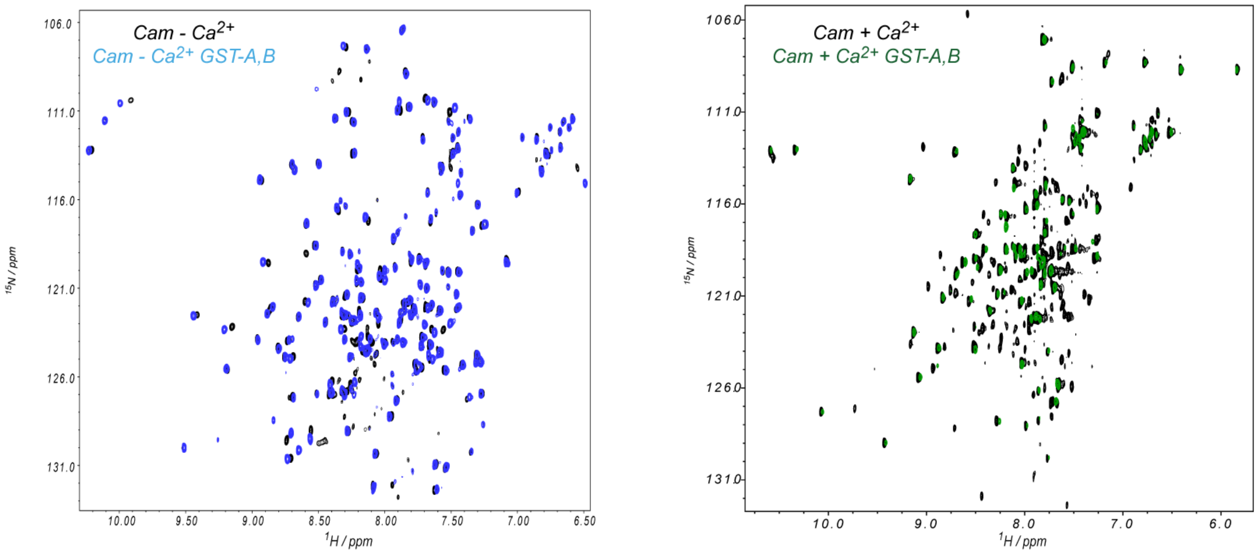

Figure S7. ^{1}H-^{15}N-HSQC spectra of calcium free (left) and calcium bound (right) calmodulin in the absence (black) and in the presence (color) of Q2AB peptide. Spectra were recorded at 298K.

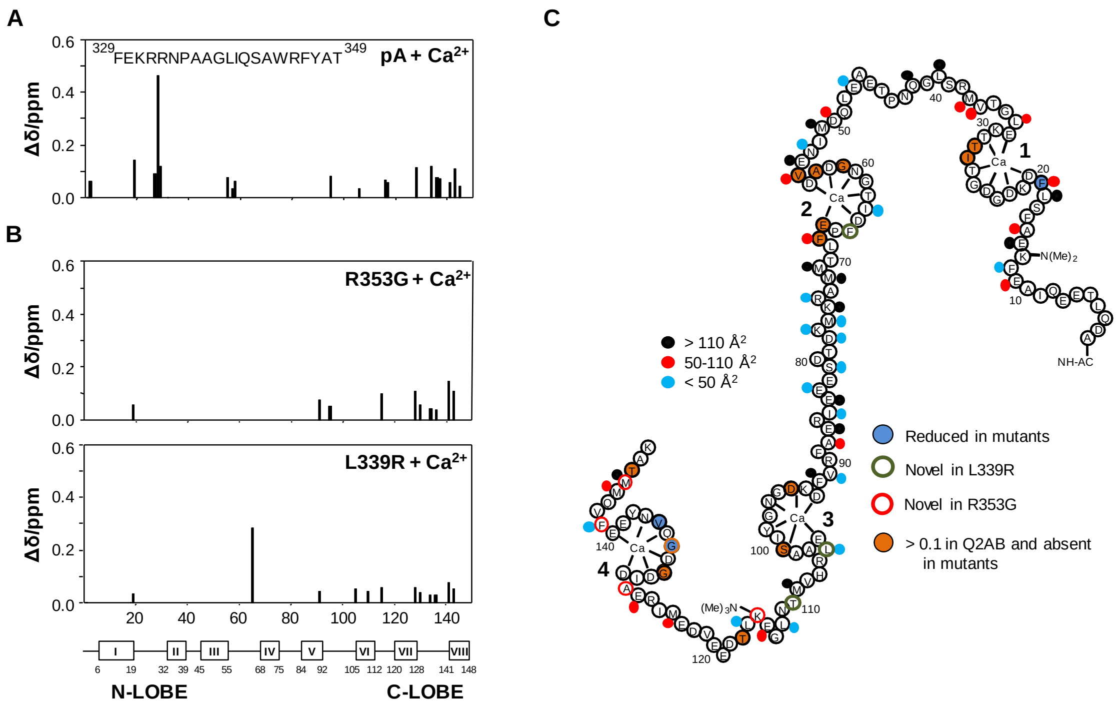

Figure S8. Monitoring the interaction between CaM and Q2AB carrying the L339R or R353G mutation by (^{1}H, ^{15}N)-HSQC spectroscopy.
(A) CaM (50 µM) CSPs induced by a peptide (250 µM) derived from the sequence of segment A plotted as a function of the CaM residues number.
(B) Partial assignment of CSPS induced by 65 µM L339R and R353G Q2AB mutants to 25 holo-CaM is plotted in function of the CaM residues number. Only residues that could be unambiguously identified are plotted. For the dataset available, the CSP maps exhibited a similar profile for both mutants.
(C) Schematic representation of CaM and the residues perturbed upon binding to Q2AB variants (Adapted from (1)). The residues are decorated with colored dots according to the interaction surface of CaM with Kv7.4 (3): blue < 50 Å^2, red > 50 Å^2 and < 110 Å^2, black > 110 Å^2. The contact surface area has been estimated using the Sobolev *et al.* algorithm (2).

References

1. Keen, J. E., R. Khawaled, D. L. Farrens, T. Neelands, A. Rivard, C. T. Bond, A. Janowsky, B. Fakler, J. P. Adelman, and J. Maylie. 1999. Domains responsible for constitutive and Ca^{2+}-dependent interactions between calmodulin and small conductance Ca^{2+}-activated potassium channels. J Neurosci. 19: 8830-8838
2. Sobolev, V., A. Sorokine, J. Prilusky, E. E. Abola, and M. Edelman. 1999. Automated analysis of interatomic contacts in proteins. Bioinformatics. 15: 327-332
3. Xu, Q., A. Chang, A. Tolia, and D. L. Minor, Jr. 2012. Structure of a Ca^{2+}/CaM:Kv7.4 (KCNQ4) B helix complex provides insight into M-current modulation. J. Mol. Biol.
